# Molecular epidemiology and antimicrobial resistance profiles of *Pseudomonas aeruginosa* causing bloodstream infections in neutropenic cancer patients

**DOI:** 10.3389/fmicb.2025.1681506

**Published:** 2025-09-30

**Authors:** Irene Cadenas-Jiménez, Ana María Badía-Tejero, Carla López-Causapé, María-Isabel Morosini, Inés Portillo-Calderón, Marina Machado, Nieves Larrosa, Piluca Martín Dávila, Zaira Palacios-Baena, Adaia Puig-Albasanz, Fe Tubau, Antonio Oliver, Enric Sastre, Sara Martí, Carlota Gudiol

**Affiliations:** ^1^Microbiology Department, Bellvitge University Hospital, Bellvitge Biomedical Research Institute (IDIBELL)-University of Barcelona, L’Hospitalet de Llobregat, Barcelona, Spain; ^2^Centre for Bomedical Research in Respiratory Diseases Network (CIBERES), ISCIII, Madrid, Spain; ^3^Department of Pathology and Experimental Therapeutics, University of Barcelona, Barcelona, Spain; ^4^Infectious Diseases Department, Bellvitge University Hospital, Bellvitge Biomedical Research Institute (IDIBELL)-University of Barcelona, L’Hospitalet de Llobregat, Barcelona, Spain; ^5^Microbiology Department, Hospital Son Espases, IdISBa, Palma De Mallorca, Spain; ^6^Centre for Biomedical Research in Infectious Diseases Network (CIBERINFEC), Instituto de Salud Carlos III, Madrid, Spain; ^7^Microbiology Department, Ramon y Cajal University Hospital, Madrid, Spain; ^8^Unit of Infectious Diseases and Clinical Microbiology, Instituto de Biomedicina de Sevilla (IBiS)/Consejo Superior de Investigaciones Científicas (CSIC), Hospital Universitario Virgen Macarena, Departamento de Microbiología, Universidad de Sevilla, Seville, Spain; ^9^Department of Clinical Microbiology and Infectious Diseases, Gregorio Marañón University General Hospital, Madrid, Spain; ^10^Microbiology Department, Vall d’Hebron University Hospital, Barcelona, Spain; ^11^Infectious Diseases Department, Ramon y Cajal Hospital, Madrid, Spain; ^12^Unit of Infectious Diseases and Clinical Microbiology, Instituto de Biomedicina de Sevilla (IBiS)/ Consejo Superior de Investigaciones Científicas (CSIC), Hospital Universitario Virgen Macarena, Departamento de Medicina, Universidad de Sevilla, Seville, Spain; ^13^Infectious Diseases Department, Vall d’Hebron Barcelona Campus Hospitalari, Barcelona, Spain; ^14^Institut Català d’Oncologia (ICO), L’Hospitalet de Llobregat, Barcelona, Spain

**Keywords:** *Pseudomonas aeruginosa*, bacteraemia, bloodstream infection, neutropenia, multidrug resistance, virulence factors, cancer

## Abstract

**Background:**

Bloodstream infections (BSI) in neutropenic cancer patients, particularly those caused by *Pseudomonas aeruginosa* (PA), are associated with high morbidity and mortality. The increasing prevalence of multidrug-resistant (MDR) and extensively drug-resistant (XDR) PA strains complicates clinical management. This study aimed to characterise PA strains causing BSI in neutropenic cancer patients and assess the association between microbiological features and clinical outcomes.

**Methods:**

We analysed PA strains from 94 BSI episodes in neutropenic cancer patients across five Spanish hospitals (2006–2018). Antimicrobial resistance, alginate and pigment production were assessed. Whole-genome sequencing was performed to identify resistance mutations and virulence genes.

**Results:**

PA strains exhibited high genetic diversity, with ST175 as the most prevalent clone (28.7%). MDR non-XDR and XDR strains accounted for 10.3% and 18.1% of cases, respectively. The highest resistance rates were for ciprofloxacin (42.6%) and imipenem (36.2%). Resistance was primarily driven by chromosomal mutations. ExoU was present in 24.4% of strains, associated with serotype O11 and ST253. Seven-day and 30-day mortality were 21.3% and 31.9%, respectively. Mortality was not significantly influenced by resistance phenotypes or the presence of ExoU. Polymicrobial infection (*p* = 0.016), septic shock (*p* < 0.001), Intensive Care Unit admission (*p* = 0.002), and inadequate empirical antibiotic therapy (*p* = 0.002), were linked to increased 7-day mortality.

**Conclusion:**

ST175 was the dominant high-risk clone, associated with antimicrobial resistance, while virulence traits were more common in susceptible strains. Inadequate empirical antibiotic therapy and septic shock significantly impacted early 7-day mortality, underscoring the need for early diagnosis and optimised treatment strategies.

## Introduction

Bloodstream infections (BSI) are among the most frequent and severe complications in neutropenic onco-haematological patients, contributing to high morbidity and mortality. The aetiology of BSI in this patient population has experienced a notable shift in recent years, with Gram-negative bacilli (GNB) emerging as the predominant causative agents in some institutions (Gustinetti and Mikulska, 2016). Among GNB, *Pseudomonas aeruginosa* (PA) has become a particularly concerning pathogen due to its capacity to cause severe and life-threatening infections, as well as its intrinsic antimicrobial resistance and ability to acquire additional resistance mechanisms (López-Causapé et al., 2018). BSI caused by multidrug-resistant (MDR) and extensively drug-resistant (XDR) PA strains are on the rise globally and are associated with poorer outcomes, especially in immunocompromised patients (Viasus et al., 2020). The severity of PA BSI is influenced by a combination of factors, including the site of infection, antibiotic treatment and microbiological determinants. Host factors, such as chemotherapy-induced neutropenia, also increase the risk of severe infection and death.

PA is recognised for its high intrinsic antimicrobial resistance (Matar et al., 2017) and its remarkable ability to acquire mutations in chromosomal genes that result in significantly increased antimicrobial resistance levels (Cortes-Lara et al., 2021). Several resistance mechanisms have been identified in PA strains, including efflux pumps, chromosomal β-lactamase production, an impermeable outer membrane, antibiotic-inactivating enzymes, and chromosomal mutations (Hu et al., 2021). Beyond this extraordinary capacity for acquiring antimicrobial resistance, the versatile metabolism of PA also provides a formidable virulence arsenal (Javanmardi et al., 2019). Multiple virulence factors have been described in PA infection. This bacterium possesses six types of secretion systems, including the type VI secretion system (T6SS), type IV secretion system (pili), and the multi-toxin component type III secretion system (T3SS). T3SS is a complex system that may severely impede host defence via the injection of cytotoxins (ExoU, ExoT, ExoS, and ExoY) (Qin et al., 2022). ExoU has been shown to be the most cytotoxic effector and is associated with early mortality in PA BSI (Peña et al., 2015).

Furthermore, PA produces alginate, which is associated with a decline in lung function, poor prognosis in respiratory infections, and higher levels of antimicrobial resistance (Malhotra et al., 2018). PA also produces two siderophores, pyoverdine and pyochelin, and the toxin pyocyanin (Gustinetti and Mikulska, 2016). Pyocyanin is a key virulence factor that promotes pathogenicity to host cells by disrupting electron transport, cellular respiration and energy metabolism (Hu et al., 2021).

Numerous epidemic PA strains have been described worldwide. For instance, ST175, ST235, and ST111 are frequent high-risk clones in Spain and are associated with MDR profiles and with the presence of the exotoxin ExoU in the case of ST235 (Fischer et al., 2020). The combination of resistance and virulence factors in PA limits therapeutic options, resulting in an urgent need for research into this field. The rising antibiotic resistance compromises treatment and increases the risk of toxicity from more aggressive therapies. For neutropenic cancer patients with PA BSI, timely administration of appropriate empiric antibiotic therapy is crucial, as even short delays can worsen clinical outcomes.

This study aims to phenotypically and genotypically characterise PA strains causing BSI in neutropenic cancer patients and to describe the association between microbiological characteristics and clinical outcomes.

## Materials and methods

### Study design, patients and setting

A microbiological analysis of PA strains was conducted using data from two international, multicentre, retrospective, observational cohorts involving PA BSI in neutropenic cancer patients (Bergas et al., 2022; Royo-Cebrecos et al., 2022). The IRONIC and ZENITH cohorts, whose study period ranged from 2006 to 2018, included adult (≥18 years) neutropenic onco-haematological patients, including haematopoietic stem cell transplant (HSCT) recipients, who were diagnosed with at least one episode of PA BSI during the study period.

For the purpose of this study, we analysed a total of 94 *P. aeruginosa* strains, each isolated from a unique patient, across five Spanish participating centres: Bellvitge University Hospital, Gregorio Marañón University Hospital, Virgen Macarena University Hospital, Ramón y Cajal University Hospital and Vall d’Hebrón University Hospital. Blood samples were initially processed at the Microbiology laboratories of each participating centre, and PA isolates were identified using conventional microbiological techniques specific to each laboratory. PA strains were sent to the Microbiology laboratory of the Bellvitge University Hospital for genotypic analysis. A representative sample of Bellvitge University Hospital was selected to ensure consistency in the distribution of strains across centres. Episodes of monomicrobial PA BSI and polymicrobial BSI where PA was one of the causative agents were included. Demographic and clinical data have been previously reported (Bergas et al., 2022; Royo-Cebrecos et al., 2022). Clinical outcomes were evaluated for each episode of PA BSI and microbiological characteristics were analysed.

### Clinical variables

Neutropenia was defined as an absolute neutrophil count below 0.5×10^9^/L, and severe neutropenia below 0.1 × 10^9^/L. Persistent BSI was identified by positive blood cultures after 48 h of adequate antibiotic therapy. Empirical therapy referred to the initiation of antimicrobials before susceptibility results became available, and inadequate empirical therapy was considered when the treatment lacked *in vitro* active antibiotics against the isolated pathogen. Combination treatment was defined as the concurrent use of two or more antibiotics.

The study used the definitions of MDR and XDR previously proposed by Magiorakos et al. (2012), instead of the more recent classification (Kadri et al., 2018). MDR non-XDR was defined as non-susceptibility to at least one agent in three or more antimicrobial categories, and XDR as non-susceptibility to at least one agent in all but two or fewer antimicrobial categories.

### Phenotypic characterization and antibiotic susceptibility

Alginate production by mucoid PA strains and the synthesis of pigments were evaluated through colony observation on Mueller-Hinton (MH) agar plates. Strains were routinely grown in Mueller-Hinton Agar (MHA) plates (BioMérieux) and incubated at 37 °C. Antimicrobial susceptibility was tested by microdilution using the automated MicroScan Walkaway system and available commercial panels (MIC57, Beckman Coulter), following the recommended clinical breakpoints of The European Committee on Antimicrobial Susceptibility Testing (EUCAST) (2023). The following antimicrobials were tested: Piperacillin-tazobactam, ceftazidime, cefepime, ceftolozane-tazobactam, ceftazidime-avivactam, aztreonam, imipenem, meropenem, ciprofloxacin, tobramycin, amikacin and colistin. To better characterize the PA population, we performed a resistotype distribution analysis. Resistance pattern distribution (resistotyping) and MAR index of PA isolates were performed in accordance with previous studies (Mendes Pedro et al., 2024).

### Whole genome sequencing and phylogenetic analysis

Short-read whole genome sequencing was performed in all the strains. DNA was extracted using the QIAamp DNA Mini Kit (Qiagen) and quantified with a Qubit 4 Fluorometer (Thermo Fisher Scientific). The libraries were prepared using the DNAprep Library Preparation Kit (Illumina) followed by paired-end sequencing (2 × 150) on a NextSeq platform (Illumina). FastQ sequences were assembled with INNUca v4.2.0 pipeline^1^ using default parameters.

MLST was determined in PA genomes using the MLST v2.4 software.^2^ Serotypes were determined in silico using PAst 1.0 from Center for Genomic Epidemiology. For phylogenetic analysis, the core-genome SNP alignment was obtained with Snippy’s core module^3^ and subjected to the prediction and removal of recombinant regions using the Gubbins v2.3.1 software (Croucher et al., 2015). Phylogenetic tree was constructed using strain PA PAO1 (NZ_CP129519.1) as reference.

### Resistance and virulence determinants

Reads were used to perform a variant calling analysis using previously defined and validated protocols (Cortes-Lara et al., 2021) to study mutations that can confer antimicrobial resistance. To determine the presence of acquired resistance genes and virulence factor, *de novo* assemblies were screened using Abricate v0.8.0^4^ with the ResFinder v3.2 database (Ferrer Florensa et al., 2022) and the Virulence Finder database (Kleinheinz et al., 2014).

### Statistical analysis

Baseline characteristics of patients were described using the median and interquartile range (IQR) for continuous variables, and frequencies for categorical variables. The Chi-squared Pearson test and Student’s *t* test were used to compare categorical and continuous variables, respectively. A significance level of *p* < 0.05 was considered statistically significant. All statistical analyses were conducted using the SPSS software package v.27.0 (SPSS Inc., Chicago, Il, USA).

## Results

### Clinical features of the study population

Ninety-four neutropenic cancer patients diagnosed with PA BSI were analysed (Table 1). Fifty-five patients (58.5%) were male, with a median age of 61 years (IQR: 54.0–72.0). The majority of patients (70.2%) had haematological malignancies, most commonly acute myeloid leukaemia (47.0%) and lymphoma (27.3%). Additionally, 19 patients (20.2%) were HSCT recipients, of whom 63.2% had undergone allogeneic transplantation. Solid tumours accounted for 29.8% of cases, with lung cancer (42.8%) and breast cancer (21.4%) being the most prevalent. The most common primary source of BSI was endogenous (41.5%), followed by pneumonia (27.7%) and perianal infection (6.4%). Septic shock was present in 29.8% of patients at diagnosis. Inadequate empirical antibiotic therapy (IEAT) was administered in 17.0% of patients, and intensive care unit (ICU) admission was required in 13.8% of them. Seven and 30-day mortality rates were 21.3 and 31.9%, respectively. Patients who died within 7 days from BSI onset had significantly higher rates of polymicrobial infection (43.8% vs. 13.0%, *p* = 0.016), septic shock (42.6% vs. 12.1%, *p* < 0.001), ICU admission (53.8 vs. 16.0%, *p* = 0.002), and IEAT (50.0% vs. 15.4%, *p* = 0.002), compared to survivors. At 30 days, polymicrobial infection (62.5 vs. 25.6%, *p* = 0.004), septic shock (57.1 vs. 21.2%, *p* < 0.001), ICU admission (61.5% vs. 27.2%, *p* = 0.014) and persistent BSI (75.0% vs. 24.4%, *p* = 0.057) were also associated with higher mortality.

**Table 1:** Clinical features and outcomes of neutropenic cancer patients with *P. aeruginosa* bloodstream infection according to resistance phenotypes and the presence of ExoU.

Characteristics	Total	Resistance phenotype			ExoU	
		Non MDR *PA*	MDR non-XDR *PA*	XDR *PA*	ExoU+	ExoU−
	(*n* = 94)	(*n* = 67)	(*n* = 10)	(*n* = 17)	(*n* = 23)	(*n* = 71)
Male patients (*n*, %)	55 (58.5)	38 (56.7)	6 (60.0)	11 (64.7)	10 (56.5)	45 (63.4)
Age (years, median, IQR)	61 (54.0–72.0)	61 (55.5–74.0)	58 (39.3–62)	59 (47–71)	61 (50–72.5)	61 (56.5–71.5)
Comorbidities (*n*, %)	39 (41.5)	29 (74.4)	3 (30.0)	7 (41.2)	9 (39.1)	30 (42.3)
Diabetes mellitus	9 (23.1)	9 (31.0)	0	0	3 (33.3)	6 (20.0)
Chronic respiratory disease	8 (20.5)	6 (20.7)	0	2 (11.8)	3 (33.3)	5 (16.7)
Chronic heart disease	5 (12.8)	5 (17.2)	0	0	1 (11.1)	4 (13.3)
Chronic kidney disease	1 (2.6)	1 (3.4)	0	0	0	1 (3.3)
Chronic liver disease	1 (2.6)	1 (3.4)	0	0	0	1 (3.3)
Underlying cancer disease (*n*, %)						
Haematological malignancy	66 (70.2)	45 (67.2)	7 (70.0)	14 (82.4)	15 (65.2)	51 (71.8)
Acute myeloid leukaemia	31 (47.0)	20 (44.4)	5 (71.4)	6 (42.9)	9 (60.0)	22 (42.3)
Lymphoma	18 (23.3)	12 (26.7)	1 (14.3)	5 (35.7)	1 (6.7)	17 (33.3)
Other haematological malignancies	17 (25.8)	13 (28.9)	1 (14.3)	3 (21.4)	5 (33.3)	12 (23.5)
Haematopoietic stem cell transplant (HSCT)	19 (20.2)	12 (63.2)	4 (40.0)	3 (17.6)	4 (17.4)	15 (21.1)
Allogeneic HSCT	12 (63.2)	6 (50.0)	3 (30.0)	3 (100)	4 (100)	8 (53.3)
Solid organ tumour	28 (29.8)	22 (32.8)	3 (30.0)	3 (17.6)	8 (34.8)	20 (28.2)
Lung cancer	12 (42.8)	9 (40.9)	1 (33.3)	2 (66.7)	4 (50.0)	8 (40.0)
Breast cancer	6 (21.4)	4 (18.2)	1 (33.3)	1 (33.3)	3 (37.5)	3 (15.0)
Nosocomial acquisition (*n*, %)	61 (64.9)	40 (59.7)	8 (80.0)	13 (76.5)	14 (60.9)	47 (66.2)
Source of BSI (*n*, %)						
Endogenous source	39 (41.5)	26 (38.8)	4 (40.0)	9 (52.9)	12 (52.2)	27 (38.0)
Respiratory tract infection	26 (27.7)	20 (29.9)	3 (30.0)	3 (17.6)	7 (30.4)	19 (26.8)
Perianal infection	6 (6.4)	6 (9.0)	0	0	2 (8.7)	4 (5.6)
Urinary tract infection	5 (5.3)	2 (3.0)	0	3 (17.6)	0	5 (7.0)
Intravascular catheter infection	5 (5.3)	2 (3.0)	2 (20.0)	1 (5.9)	1 (4.3)	4 (5.6)
Skin and soft tissue infection	4 (4.3)	2 (3.0)	1 (10.0)	1 (5.9)	0	4 (5.6)
Neutropenic enterocolitis	3 (3.2)	3 (4.5)	0	0	0	3 (4.2)
Other intra-abdominal infection	3 (3.2)	3 (4.5)	0	0	0	3 (4.2)
Unknown	3 (3.2)	3 (4.5)	0	0	1 (4.3)	3 (4.2)
Polymicrobial infection (*n*, %)*	16 (17.0)	15 (22.4)	0	1 (5.9)	4 (17.4)	12 (16.9)
Septic shock at presentation (*n*, %)	28 (29.8)	20 (29.9)	1 (10.0)	7 (41.2)	7 (30.4)	21 (29.6)
Antibiotic therapy and outcomes (*n*, %)						
Persistent bacteraemia (48 h)	4 (4.3)	3 (4.5)	0	1 (5.9)	1 (4.3)	3 (4.2)
ICU admission	13 (13.8)	7 (10.4)	1 (10.0)	5 (29.4)	2 (8.7)	11 (15.5)
Inadequate empirical antibiotic therapy	16 (17.0)	3 (4.5)	4 (40.0)	8 (47.1)	1 (4.3)	10 (14.1)
7-day mortality	20 (21.5)	16 (23.9)	1 (10.0)	3 (17.6)	4 (17.4)	16 (22.5)
30-day mortality	30 (32.3)	25 (37.3)	2 (20.0)	3 (17.6)	8 (34.8)	22 (31.0)

*Escherichia coli (*n* = 9), *Klebsiella pneumoniae* (*n* = 3), *Enterobacter cloacae* (*n* = 2), *Acinetobacter pittii* (*n* = 1), *Bacteroides* spp. (*n* = 1), *Enterococcus faecalis* (*n* = 1), *Escherichia fergusonii* (*n* = 1), *Hafnia halvei* (*n* = 1), *Serratia Marcescens* (*n* = 1), *Staphylococcus aureus* (*n* = 1), *Streptococcus gallolyticus* (*n* = 1).

MDR, multidrug resistant; XDR, extensively drug resistant; HSCT, haematopoietic stem cell transplant; ICU, intensive care unit.

### PA sequence type and antimicrobial resistance

**Figure 1:**
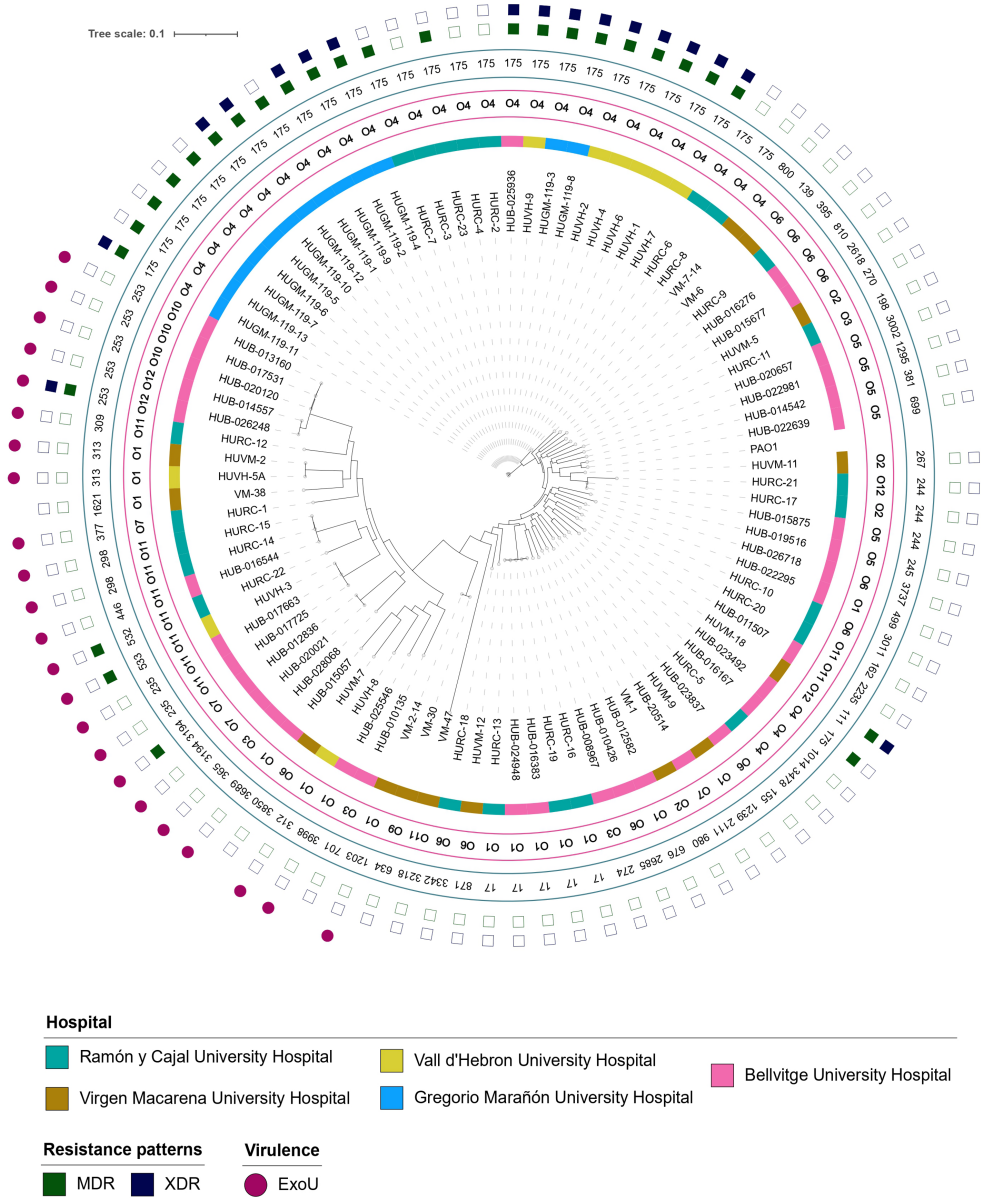
Phylogenetic tree of the 94 Pseudomonas aeruginosa strains included in the study. Hospitals, serotypes, MLSTs, resistance profiles (MDR and XDR) and presence of ExoU protein are shown.

**Figure 2:**
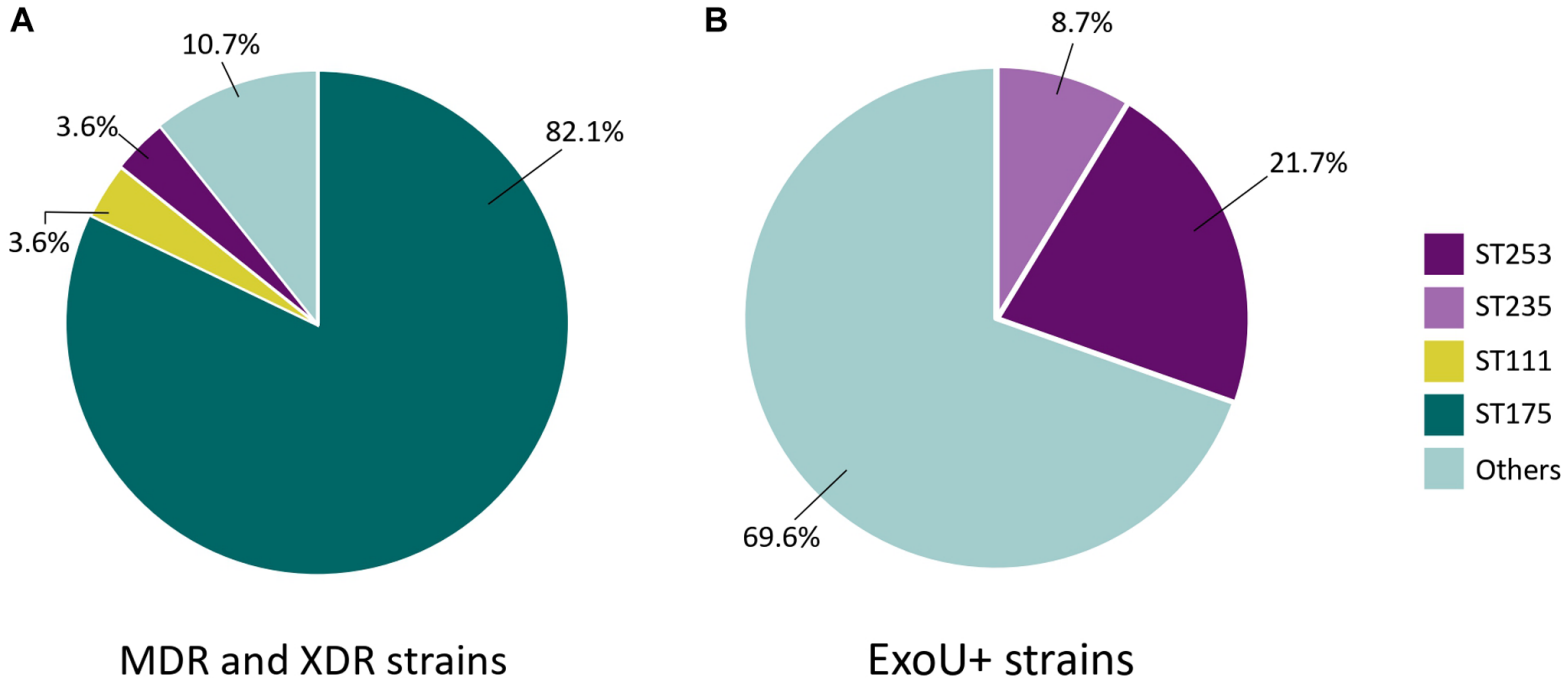
Multidrug-resistance, extensively-drug resistance and virulence phenotypes in relation with sequence types among all the 94 *Pseudomonas aeruginosa* strains selected in the study.

A total of 94 PA strains were isolated and characterised. The number of isolates provided by hospitals can be consulted in Supplementary Table S1. The strain collection showed high genetic diversity, with a total of 52 different STs (Figure 1). The high-risk clone ST175 was the most prevalent and was found in 28.7% (*n* = 27) of the strains. ST253 was found in 5.3% (*n* = 5) of the strains and was exclusively isolated in Bellvitge University Hospital. Two more high-risk STs were found in the collection but were less prevalent: ST235 (*n* = 2) and ST111 (*n* = 1) (Figure 2). Regarding silico serotypes, the most frequent was O4 (*n* = 31), followed by O1 (*n* = 14) and O11 (*n* = 11). The serotype O4 was present in all ST175 strains, while serotypes O1 and O11 showed more variability regarding sequence types.

Forty-seven strains (49%) were susceptible to all antimicrobials tested. MDR non-XDR and XDR strains were mostly related to the high-risk clone ST175 across all participating hospitals (Figure 2). Antimicrobial susceptibility patterns for all the PA strains are shown in Table 2. Overall, 10.6% (*n* = 10) were caused by MDR strains and 18.1% (*n* = 17) were caused by XDR strains. The highest resistance rates were found for ciprofloxacin (42.6%), followed by imipenem (36.2%). Resistance to cefepime (26.6%) and tobramycin were also high (30.9%), while resistance to amikacin remained low (3.2%). Additionally, one strain (1.1%) showed resistance to the more recently available antibiotics ceftolozane-tazobactam and ceftazidime-avibactam. No colistin resistance was detected in our collection.

**Table 2:** Antimicrobial resistance rates among *P. aeruginosa* strains regarding high-risk clones and resistance and virulence phenotypes.

Antimicrobial agent	Total (*n* = 94)	ST175 (*n* = 28)	ST235 (*n* = 2)	ST253 (*n* = 5)	ST111 (*n* = 1)
Piperacillin-tazobactam	16 (17%)	13 (46.4%)	0 (0%)	1 (20%)	1 (100%)
Ceftazidime	15 (16%)	12 (42.9%)	0 (0%)	1 (20%)	1 (100%)
Cefepime	25 (26.6%)	20 (71.4%)	0 (0%)	1 (20%)	1 (100%)
Ceftolozane-tazobactam	1 (1.1%)	0 (0%)	0 (0%)	1 (20%)	0 (0%)
Ceftazidime-avibactam	1 (1.1%)	0 (0%)	0 (0%)	1 (20%)	0 (0%)
Aztreonam	7 (7.4%)	3 (10.7%)	0 (0%)	0 (0%)	1 (100%)
Imipenem	34 (36.2%)	22 (78.6%)	0 (0%)	1 (20%)	1 (100%)
Meropenem	22 (23.4%)	17 (60.7%)	0 (0%)	1 (20%)	1 (100%)
Ciprofloxacin	40 (42.6%)	26 (92.9%)	0 (0%)	2 (40%)	1 (100%)
Tobramycin	29 (30.9%)	26 (92.9%)	0 (0%)	1 (20%)	1 (100%)
Amikacin	3 (3.2%)	0 (0%)	0 (0%)	1 (20%)	1 (100%)
Colistin	0 (0%)	0 (0%)	0 (0%)	0 (0%)	0 (0%)
MDR	10 (10.6%)	8 (28.6%)	0 (0%)	0 (0%)	0 (0%)
XDR	17 (18.1%)	15 (53.6%)	0 (0%)	1 (20%)	1 (100%)
ExoU	23 (24.5%)	0 (0%)	2 (100%)	5 (100%)	0 (0%)

A total of twenty-one distinct resistotypes were identified in our PA collection (Supplementary Table S2), indicating significant diversity among the various resistotypes. The predominant resistotype observed was the combined resistance to piperacillin-tazobactam, cefepime, ceftazidime, imipenem, meropenem, ciprofloxacin and tobramycin (*n* = 7, 7.4%), followed by resistance only to ciprofloxacin (*n* = 6; 6.4%).

### PA antimicrobial resistance mechanisms

**Figure 3:**
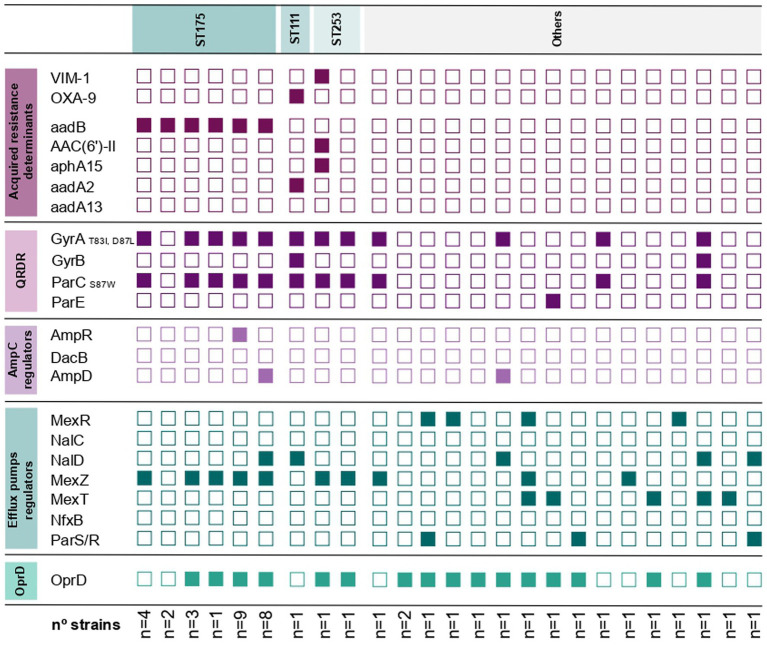
Antimicrobial resistance determinants present in all the resistant Pseudomonas aeruginosa strains isolated from neutropenic patients. Polymorphisms not related with antimicrobial resistance have been removed (Cortes-Lara et al., 2021).

Resistance to ciprofloxacin was mainly attributed to alterations in DNA gyrase and topoisomerase IV by modifications in GyrA/ParC (33/40; 82.5%). In the remaining strains, resistance to quinolones was explained by mutations in regulator genes of efflux pumps (Figure 3). Moreover, mutations in genes involved in the expression of the chromosomal β-lactamase AmpC were the most common mechanism associated with cephalosporin resistance (17/25; 68%). Two strains acquired β-lactamases via horizontal gene transfer, with VIM-1 (*n* = 1) being associated with the high-risk clone ST253, and the β-lactamase OXA-9 (*n* = 1) with the high-risk clone ST111. The strain harbouring the β-lactamase VIM-1 was the only one resistant to the more recently available antibiotics ceftolozane-tazobactam and ceftazidime-avibactam. Resistance to both imipenem and meropenem was primarily driven by inactivation of the OprD porin. In contrast, only two strains were imipenem resistant due to the acquisition of a broad-spectrum β-lactamase. Additionally, meropenem resistance frequently involved overexpression of the MexXY-OprM efflux pump. Finally, aminoglycoside resistance was mostly attributed to the acquisition of aminoglycoside-modifying enzymes (27/29; 93.1%), with *aadB* enzyme being the most frequent and associated with the high-risk clone ST175 (*n* = 28). The remaining aminoglycoside-resistant strains exhibited mutations affecting the expression of the MexXY-OprM efflux pump.

### PA virulence

The exotoxin ExoU was identified in 24.5% of the strains (Table 2) and was related to the high-risk clones ST253 (5/23, 21.7%) and ST235 (2/23, 8.7%) (Figure 2B). Additionally, the serotype O11 was the most frequent serotype found among ExoU-producing strains (8/11 of O11 strains, 72.7%), and was related to a variety of different STs, including the high-risk clone ST235. Notably, ST253 also carried ExoU and was exclusively associated with serotypes O12 and O10. Strains with a more virulent phenotype presented low antimicrobial resistance rates. Interestingly, one ST253 PA strain presented the exotoxin ExoU and an XDR phenotype. Mucoid phenotype was not frequent among our collection (*n* = 1; 1.1%). Regarding visible pigment production, pyoverdine was the most frequently found (*n* = 10; 10.6%), followed by pyocyanin (*n* = 2; 2.1%) and pyochelin (*n* = 1; 1.1%). The presence of these phenotypes was not correlated with any specific clone.

### Association of microbiological features with clinical outcomes

The high-risk clone ST175 was predominantly associated with MDR non-XDR (8/28; 28.6%) and XDR phenotypes (15/28; 53.6%) (Table 2). PA BSI caused by MDR non-XDR or XDR strains was linked to a higher rate of IEAT compared to susceptible strains (*p* = 0.001 and *p* < 0.001, respectively) (see Tables 1, 3). Infection due to XDR strains was also significantly associated with ICU admission when compared with susceptible strains (*p* = 0.046). In contrast, no significant differences in 7-day mortality were observed between susceptible and MDR non-XDR strains (*p* = 0.443), nor between XDR and susceptible (*p* = 0.751). Similarly, 30-day mortality did not differ significantly between MDR non-XDR strains and susceptible strains (*p* = 0.479), or between XDR and susceptible strains (*p* = 0.157). Additionally, no association was observed between the presence of ExoU and 7 and 30-day mortality (*p* = 0.772 and *p* = 0.734, respectively).

**Table 3:** Empirical antibiotic therapy according to resistance phenotypes and outcomes.

Antimicrobial treatment	Total (*n* = 94)	MDR non-XDR PA (*n* = 10)	XDR PA (*n* = 17)	7-day mortality (*n* = 20)	30-day mortality (*n* = 30)
*Empirical treatment (n, %)*					
Combined therapy	47 (50.0)	4 (10.0)	7 (41.2)	7 (35.0)	13 (43.3)
Antipseudomonal β-lactam + aminoglycoside	45 (95.7)	3 (75.0)	7 (100)	7 (100)	13 (100)
Antipseudomonal β-lactam + other	2 (4.3)	1 (25.0)	0	0	0
Monotherapy	44 (46.8)	6 (60.0)	8 (47.1)	11 (55.0)	15 (50.0)
Piperacillin/tazobactam	27 (61.4)	2 (33.3)	5 (62.5)	4 (36.4)	6 (40.0)
Antipseudomonal cefalosporins	5 (11.4)	3 (50.0)	2 (25.0)	1 (9.1)	2 (13.3)
Antipseudomonal carbapenems (imipenem, meropenem)	11 (25.0)	1 (16.7)	1 (12.5)	6 (54.5)	7 (46.7)
Quinolones	1 (2.3)	0	0	0	0
Did not receive antipseudomonal therapy	3 (4.3)	0	1 (5.9)	2 (10.0)	2 (6.7)
*Adequate empirical treatment (n, %)*	78 (83.0)	6 (60.0)	9 (52.9)	12 (60.0)	22 (73.3)
Combined therapy	47 (60.3)	4 (66.7)	7 (77.8)	7 (58.3)	13 (59.1)
Antipseudomonal β-lactam + aminoglycoside	45 (95.7)	3 (75.0)	7 (100)	7 (100)	13 (100)
Antipseudomonal β-lactam + other	2 (4.3)	1 (25.0)	0	0	0
Monotherapy	31 (39.7)	2 (33.3)	2 (22.2)	5 (41.7)	9 (40.9)
Piperacillin/tazobactam	22 (71.0)	1 (50.0)	1 (50.0)	3 (60.0)	5 (55.6)
Antipseudomonal cefalosporins	1 (3.2)	1 (50.0)	0	0	1 (11.1)
Antipseudomonal carbapenems (imipenem, meropenem)	7 (22.6)	0	1 (50.0)	2 (40.0)	3 (33.3)
Quinolones	1 (3.2)	0	0	0	0
*Inadequate empirical treatment (n, %)*	16 (17.0)	4 (40.0)	8 (47.1)	8 (40.0)	8 (26.7)
Combined therapy	0	0	0	0	0
Monotherapy	13 (81.3)	4 (100)	6 (75.0)	6 (75.0)	6 (75.0)
Piperacillin/tazobactam	5 (38.5)	1 (25.0)	4 (66.7)	1 (16.7)	1 (16.7)
Antipseudomonal cefalosporins	4 (30.8)	2 (50.0)	2 (33.3)	1 (16.7)	1 (16.7)
Antipseudomonal carbapenems (imipenem, meropenem)	4 (30.8)	1 (25.0)	0	4 (66.7)	4 (66.7)
Quinolones	0	0	0	0	0
Did not receive antipseudomonal therapy	3 (3.2)	0	2 (25.0)	2 (25.0)	2 (25.0)

## Discussion

The interplay between antimicrobial resistance and virulence factors of PA and their potential impact on clinical outcomes has become a subject of growing interest. In this study, we provide a detailed phenotypic and genotypic characterization of PA strains from a cohort of neutropenic cancer patients with PA BSI. Additionally, we assessed how these microbiological traits could influence clinical outcomes and prognosis.

Our PA collection exhibited significant genetic diversity, with the presence of several high-risk clones, the most prevalent being ST175. Previous studies in Spain have also reported ST175 as the most prevalent high-risk clone, followed by ST235 (del Barrio-Tofiño et al., 2019; Sastre-Femenia et al., 2023). However, in our study, ST235 was less common, with ST253 emerging as the second most frequent high-risk clone. The detection of high-risk clones in immunocompromised patients is particularly concerning, as they are often associated with increased resistance and/or enhanced virulence (Oliver et al., 2015; del Barrio-Tofiño et al., 2020). ST175 was linked to MDR non-XDR and XDR phenotypes. Most ST253 strains are known for their high antimicrobial susceptibility and increased virulence, primarily linked to ExoU (Fischer et al., 2020). Our study identified one ST253 strain which displayed both the presence of ExoU and an XDR phenotype, which is a less common combination of resistance and virulence. This finding aligns with a recent report from Spain, where ST253 strains exhibiting XDR characteristics were also documented (Arca-Suárez et al., 2021).

Analysis of antimicrobial resistance in our study revealed a high prevalence of MDR-non XDR and XDR phenotypes, which were resistant to most antipseudomonal agents, except for colistin and newer combinations of ceftolozane/tazobactam and ceftazidime/avibactam, in agreement with previous studies (Sastre-Femenia et al., 2023; Bogiel et al., 2022; Cabot et al., 2016). Notably, the rate of extended-spectrum β-lactamase/carbapenemase production was low (2.1%), consistent with the decreasing prevalence of these resistance mechanisms reported in a recent Spanish surveillance study (Sastre-Femenia et al., 2023). Aminoglycoside-modifying enzymes were the most commonly acquired antimicrobial resistance enzymes in the collection, predominantly associated with the high-risk clone ST175, as previously reported (Recio et al., 2020). For the remaining strains, resistance was attributed to chromosomal point mutations, which are among the most frequently reported in PA (López-Causapé et al., 2018). These include modifications in GyrA and ParC, primarily responsible for quinolone resistance, as well as alterations in OprD and AmpC, associated with β-lactam resistance. Efflux pump overexpression emerged as a frequent resistance mechanism, with MexXY overexpression being particularly prevalent, often linked to alterations in the *mexZ* regulator gene. In contrast, no overexpression of MexAB-OprM was detected, which is consistent with previous findings in Spain showing that common mutations in its regulatory genes often represent natural polymorphisms without functional impact on expression levels (Cabot et al., 2016). Furthermore, among the common mutations of the ST175 lineage, we observed the characteristic G154R substitution in AmpR and the truncating Q142X modification in OprD, though their presence varied among isolates. In contrast, the universal ST175 markers (including GyrA/ParC mutations and the MexZ G195E variant) were consistently present in all strains except in two atypical strains. The uniformity of these mutations is intrinsic to the ST175 clone and has been reported globally, independently of geographic or institutional origin (Cabot et al., 2016).

In accordance with our findings, mortality due to *P. aeruginosa* BSI in neutropenic cancer patients is recognized to be high (Gustinetti and Mikulska, 2016). We observed that 7-day mortality was higher among patients who received IEAT, had a polymicrobial infection, or developed septic shock. The impact of septic shock and polymicrobial infection persisted at 30 days, and persistent bacteraemia was also associated with decreased survival at this time point. No significant differences in mortality were observed according to antibiotic resistance phenotypes at 7 and 30 days.

These results suggest that antimicrobial resistance alone may not be the primary determinant of survival in this patient population. Disease severity, septic shock, and appropriate empirical antibiotics are key determinants of survival in neutropenic cancer patients (Gudiol et al., 2020). Early recognition and aggressive management of PA BSI are crucial. Although resistance profiles may not directly increase mortality, they can delay effective treatment, highlighting the need for optimized empirical strategies.

The pathogenicity of PA is influenced not only by specific virulence factors but also by the T3SS genotype. Among these, the ExoU toxin stands out as a key contributor to virulence and has been linked to unfavourable outcomes in both experimental and clinical research (Peña et al., 2015; Recio et al., 2018; El-Solh et al., 2012). A Spanish multicenter study (Recio et al., 2018) reported ExoU-production as an independent risk factor for early mortality in PA BSI. However, in our cohort we did not observe a significant association between the presence of *exoU* and mortality, suggesting that other factors may play a more prominent role in determining patient outcomes.

A key limitation of this study is its retrospective design, which may introduce biases and limit the ability to establish causal relationships. Additionally, while this was a multicentre study, our PA strain collection reflects only the phenotypic and genetic characteristics of isolates from five hospitals in Spain. The findings may not be generalizable to other regions, where the prevalence of specific strains may differ. Moreover, the sample size of the molecularly characterized subset may limit the ability to detect certain associations with clinical outcomes. Nevertheless, the trends observed provide valuable insights and generate hypotheses that warrant further investigation in larger cohorts. Despite these limitations, this study provides a comprehensive phenotypic and genotypic characterisation of PA strains causing BSI in high-risk neutropenic cancer patients. By integrating microbiological data with clinical outcomes, it contributes significantly to the broader understanding of PA infections in high-risk neutropenic patients and serves as a foundation for future research into this field.

## Conclusion

Our findings highlight the significant genetic diversity among PA strains causing BSI in neutropenic cancer patients, with ST175 emerging as the predominant high-risk clone. While the prevalence of MDR non-XDR and XDR phenotypes remains high, we observed a slight decline in extended-spectrum β-lactamase/carbapenemase production, which may have implications for future resistance trends. Our results also underscore the need for optimised empirical antibiotic strategies in patients at risk for PA infection. Future research should focus on larger, prospective, multicentre studies to further explore the complex interplay between antimicrobial resistance, virulence factors, and clinical management strategies for PA BSI in neutropenic cancer patients.

## Data Availability

The datasets presented in this study can be found in online repositories. The names of the repository/repositories and accession number(s) can be found in the article/Supplementary material.

## Footnotes

^1^ https://github.com/B-UMMI/INNUca

^2^ https://github.com/tseemann/mlst

^3^ https://github.com/tseemann/snippy

^4^ https://github.com/tseemann/abricate
